# Social overload and fear of negative evaluation mediate the effect of neuroticism on classroom disruptions that predicts occupational problems in teachers over two years

**DOI:** 10.1007/s11218-025-10024-w

**Published:** 2025-02-05

**Authors:** Gabriel Jenni, Alexander Wettstein, Ida Schneider, Fabienne Kühne, Martin grosse Holtforth, Roberto La Marca

**Affiliations:** 1 Department of Research and Development, University of Teacher Education Bern, Fabrikstrasse 8, 3012 Bern, Switzerland; 2 Clinical Psychology and Psychotherapy, Department of Psychology, University of Bern, Bern, Switzerland; 3 Psychosomatic Medicine, Department of Neurology, Inselspital, Bern University Hospital, University of Bern, Bern, Switzerland; 4 Clinica Holistica Engiadina, Susch, Switzerland; 5 Clinical Psychology and Psychotherapy, Department of Psychology, University of Zurich, Zurich, Switzerland

**Keywords:** Classroom disruptions, Behavioral observation, Teacher stress, Neuroticism, Fear of negative evaluation, Social overload

## Abstract

Research on teacher stress has identified classroom disruptions as a major risk factor. However, teachers perceive classroom disruptions “through the lens” of their own personalities. This observational study involving 42 teachers (28 female, *M*age = 39.66, *SD* = 11.99) aimed to compare teacher-perceived and observed classroom disruptions and examine how neuroticism, fear of negative evaluation, and social overload influence teachers’ perception of classroom disruptions. Moreover, longitudinal associations between perceived and observed classroom disruptions and occupational problems were investigated over two years. Results show that neuroticism, fear of negative evaluation, and social overload are positively associated with more perceived classroom disruptions. In this context, neuroticism does not directly lead to more perceived classroom disruptions, but the relationship is mediated by fear of negative evaluation and social overload. Moreover, perceived classroom disruptions were associated with an increase of occupational problems over two years. Examining self-reports in combination with behavioral observation is crucial for better understanding teachers’ perception of classroom disruptions and identifying the personality traits and social stressors influencing perception for preventing teachers’ health problems. Teacher education must support teachers in dealing with fear of negative evaluation and social overload and recognize the longitudinal cumulative effects of biased perception on teacher stress. This can prevent teachers from leaving the profession early and keep burnout rates and costs low.

## Introduction

Student misbehavior is recognized as a major source of stress for teachers (Boyle et al., 1995; Byrne, 1999; Dicke et al., 2014; Evers et al., 2004; McCormick & Barnett, 2011). However, teachers themselves may also disrupt classroom processes (Wettstein & Scherzinger, 2022). The present study focuses on *classroom disruptions* as interruptions in the teaching–learning process (Scherzinger & Wettstein, 2019). As such, classroom disruptions can emanate from students, the teacher, or external intrusions. However, the term classroom disruptions does not refer to individual behavior but to the reciprocal behavior of the participants in the interaction. Therefore, we define classroom disruptions as interactional processes that hinder teaching and learning.

Both students and teachers can contribute to these interactional disturbances through individual behavior. Student disruption (e.g., agitation, cutting in, or threatening) hampers teaching effectiveness and decreases the opportunities for disruptive students and their classmates to learn. Teachers might further contribute to classroom disruptions by reacting inappropriately (e.g., shaming or ridiculing), thus aggravating an already problematic situation (Scherzinger & Wettstein, 2022). An observational study showed that teachers disrupt lessons as often as individual students by arriving late, organizing the classroom poorly, interrupting periods of concentrated work, or embarrassing students (Scherzinger & Wettstein, 2019). The extension of classroom disruptions over the entire lesson setting can lead to a learning environment characterized by a lack of concentration and restlessness (Skiba & Rausch, 2006).

Classroom disruptions affect both students and teachers negatively. They were shown to be positively associated with biological stress mediators (La Marca et al., 2023) and have unfavorable impact on teacher health (Ingersoll, 2001, 2003), thus potentially reducing teaching quality, student motivation, and student performance (Blank & Shavit, 2016; Klusmann et al., 2016; Shen et al., 2015). Persisting classroom disruptions can further increase teachers’ occupational problems, which include emotional, cognitive, and behavioral problems. In the long run, these problems affect teachers’ work performance, mental and physical health, and social well-being, potentially resulting in burnout (Bellingrath et al., 2008; García-Carmona et al., 2019). Given these far-reaching consequences, it is important to understand classroom disruptions and identify underlying factors to prevent occupational problems in teachers.

## Teachers’ perception of classroom disruptions is subjective

Teachers do not perceive classroom disruptions objectively but are influenced by their personal characteristics and social context. Multi-informant studies combining teacher ratings and systematic behavioral observation found only weak (*r* = 0.30, Skiba, 1989) to moderate (*r* = 0.51, Scherzinger & Wettstein, 2019) correlations between observed and teacher-perceived classroom disruptions. Thus, teachers’ perception rarely matches the perception of observers. Rather, their perception is subjective and characterized by either over- or underestimation in relation to observations. In contrast, external observers can only assess a teacher’s actual behavior and are not influenced by possible intentions, goals, or justifications that may guide the behavior and could influence teachers’ perception (Pronin & Kugler, 2007). Observers can compare different classes and make comprehensible judgments guided by rules; they are not involved in the interaction and can, therefore, take an external perspective that approaches “objectivity” (Praetorius et al., 2012).

Wubbels et al. (1992) compared teachers’ and students’ ratings of teachers’ interpersonal behavior (e.g., teacher-student relationship) and found that most teachers are too optimistic and overestimate their own interpersonal behavior. However, a considerable group of teachers also underestimated their interpersonal behavior compared to students’ ratings. These teachers perceive themselves to be worse than they are. Generally, an optimistic view of the world and one’s abilities is seen as adaptive. Thus, underestimating the occurrence of classroom disruptions can be seen as a coping strategy to protect one’s self-esteem. However, overestimating classroom disruptions could also be seen as protecting a teacher’s self-esteem in the short term by preparing for anticipated failure or disappointment (e.g., self-handicapping). It is not yet clear what leads to this perception that differs from behavioral observations and which consequences arise.

Teachers’ perception can be influenced by several factors. First, teaching is inherently social. Teachers are constantly interacting with students, colleagues, administration, and parents. Classroom interactions are highly complex due to high social density, simultaneity, immediacy, unpredictability, informality, and publicity (Doyle, 2006; Herzog, 2006; Lortie, 2002). Thus, teachers might observe classroom processes less accurately than observers because they deal with complex social situations and must multitask. Second, teacher characteristics such as personality traits might influence their perceptions. A previous publication from our group based on the present sample showed that teachers’ chronic worries and resignation lead to an overestimation of student aggression and more vital exhaustion (Wettstein et al., 2023b). Third, the high social-interactional demands of the teaching profession combined with specific risk factors could further boost the relationship between teacher characteristics and perception.

## Teacher characteristics can influence perception

Studies show that *neuroticism* is a crucial risk factor for increased perception of student misbehavior (Kokkinos et al., 2005) and teacher stress (Kim et al., 2019; Kokkinos, 2007). Neuroticism is a personality trait characterized by higher vulnerability to psychological stress, a tendency towards unrealistic beliefs, and difficulties with impulse control and stress-coping (Costa & McCrae, 1992). Teachers high in neuroticism may also be more likely to experience burnout due to challenging student behavior, self-doubt, and work-related rumination (McIlveen & Perera, 2016). Moreover, neurotic individuals displayed a strong negative bias when evaluating everyday events in the presence of other people compared to being alone (Uziel, 2015). Teachers are constantly exposed to social interactions, which could further aggravate the negative effects of neuroticism. However, it remains largely unclear through which specific processes neuroticism influences teachers’ perception of classroom disruptions. Two factors that may mediate the relationship between neuroticism and classroom disruptions are fear of negative evaluation (FNE) and social overload.

*FNE* describes a personality trait manifesting in social situations, characterized by fear of others’ evaluations, expectations that others will evaluate oneself negatively, as well as an associated distress (Watson & Friend, 1969). FNE may affect perceptions of stress and may lead to negative reactions (Leary et al., 1998; Shafique et al., 2017). To the best of our knowledge, there are no studies assessing FNE among teachers. However, FNE could be an important risk factor influencing teachers’ perception due to the social nature of teaching. Teacher’s actions take place in public (e.g., in front of a class), and thus, they are constantly evaluated in everyday situations by pupils, parents, and supervisors. Individuals high in FNE generally show hypervigilant-avoidance-related behavior (i.e., are overly vigilant to and then avoid threatening stimuli) when presented with emotional facial expressions (Rossignol et al., 2013; Wieser et al., 2009). Thus, it may be that teachers with high FNE are hypervigilant about negative student expressions and, in turn, overestimate disruptive behaviors. Moreover, using event-related brain potentials assessed by EEGs, Zhang et al. (2022) found that individuals high in FNE are more attentive to negative compared to positive social feedback, as opposed to individuals low in FNE. This attentional bias could also lead to more perceived classroom disruptions for teachers high in FNE. Furthermore, classroom disruptions could foster a negative self-image (e.g., losing authority), making the teacher feel inferior. Subsequently, classroom disruptions might be perceived as more dramatic.

Neuroticism and FNE are highly correlated (Allan et al., 2017). A high negative affect caused by neuroticism (i.e., the tendency to experience more negative emotional reactions and interpret information in a more negatively biased manner) could reinforce an individual’s FNE. Affected teachers might also focus more strongly on negative cues in social situations, leading them to overestimate the extent of classroom disruptions.

Many complex and ever-present interactional processes in the classroom may also lead to social overload for teachers. *Social overload* can be described as being overwhelmed by social interactions and demands (i.e., perceiving that too much time and attention is required to maintain relationships; see Zhang et al., 2016). Exposure to too many social interactions in a short time prevents one from adequately coping with the demands of the social environment, leading to stress and emotional exhaustion (Maier et al., 2012). Indeed, teachers who feel that they invest more in students, colleagues, or school than they receive in return may experience more emotional and occupational problems (van Horn et al., 2001).

This might be especially true for emotionally unstable teachers. Lo (2019) showed that neurotic social network users feel more socially overloaded than emotionally stable users. Additionally, social overload can lead to an exhaustion of mental resources (Zhang et al., 2016), which in turn could result in a lower tolerance threshold, over-sensitization, and a negative perception of social interactions. Thus, teachers high in neuroticism might experience more social overload and, in turn, perceive more classroom disruptions.

## Present study

This study is part of a larger ambulatory assessment project on stressful classroom interactions (La Marca et al., 2023; Wettstein et al., 2021, 2023a). An ambulatory assessment allows for capturing teachers’ real-life working situations. Thereby, it enhances ecological validity and our understanding of the complex interplay between psychological and social factors. So far, research on classroom disruptions is mainly based on self-reports measuring subjective perceptions. Research is still needed to understand how teachers perceive classroom disruptions, how their perception relates to observers’ assessment of the same classroom, and which factors influence this. In particular, it is unclear how neuroticism, FNE, and social overload influence teachers’ perception of classroom disruptions. Additionally, it is unclear how classroom disruptions and occupational problems are associated in the long term.

The present study aims to fill these gaps by combining teachers’ self-reports and behavioral observation using a longitudinal design. Based on the reported research, we expect only a weak to moderate association between teachers’ perception and external observers’ assessments of classroom disruptions (hypothesis 1). Moreover, we expect teachers’ self-rated characteristics (i.e., neuroticism, FNE, social overload) to be associated with teacher-perceived but not with observed classroom disruptions (hypothesis 2). We assume that neuroticism is associated with more perceived classroom disruptions, and we expect this relationship to be mediated by FNE and social overload (hypothesis 3). Moreover, we expect that an overestimation of classroom disruptions is positively associated with high neuroticism, high FNE, high social overload (hypothesis 4a), and more occupational problems (hypothesis 4b). Longitudinally, we expect that perceived but not observed classroom disruptions are associated with occupational problems in the long term (hypothesis 5).

## Method

### Participants

The sample included 42 Swiss teachers (28 female, *M*age = 39.66, *SD* = 11.99, range = 23–63) at baseline. On average, they had 13.35 years of teaching experience (*SD* = 11.07, range = 1–40). After the first measurement, one teacher moved abroad, and two teachers withdrew their participation due to pregnancy, resulting in a sample of 39 teachers one and two years later. Participants were recruited via circular emails and flyers. Inclusion criteria for participation were a minimum of 16 lessons per week (equivalent to at least 60 percent of full-time employment) and employment as a primary or secondary teacher in the canton of Bern. Enrolled teachers signed a declaration of consent. The study was approved by the ethics committee of the canton of Bern and by the Internal Review Board (IRB) of the University of Bern and was conducted in strict compliance with current data protection laws and in accordance with the Declaration of Helsinki.

### Design

This longitudinal ambulatory assessment study relies on an ecological approach to study the participants in their “natural habitats” (Trull & Ebner-Priemer, 2013), aiming to increase ecological validity. The study included three measurement points (baseline, 1-year follow-up, 2-year follow-up). Combining self-reports and observation represents a multimodal approach and reduces the common method bias.

### Procedure

At the first measurement point (t0), teachers provided information on demographic variables (sex, age, years of teaching experience, number of lessons taught per week). Participants completed online questionnaires in German on classroom disruptions, neuroticism, FNE, social overload, and occupational problems. After, research assistants visited the participants’ schools to record four consecutive lessons of 45 min each. The time delay between filling out the questionnaires and the video recordings was *M* = 189.95 days (*SD* = 86.40). One (t1) and two years (t2) later, occupational problems were assessed again.

### Measures

#### Self-reports

Perceived classroom disruptions were rated on a Likert scale from 1 (never) to 4 (very often) with four items from the classroom questionnaire (Wettstein et al., 2016). A sample item is *“There are many disruptions in my class”* and Cronbach’s *α* was 0.71*.*

Neuroticism was measured with two items of the short version of the Big Five personality trait scale (BFI-10; Rammstedt, 2007) and two additional items with the highest factor loadings of the NEO-FFI (Borkenau & Ostendorf, 1993). The structure of the combined items was tested using exploratory factor analysis. Both Bartlett’s test (*p* < 0.001) and the Kaiser–Meyer–Olkin measure of sampling adequacy (KMO = 0.675) indicated that the variables are suitable for factor analysis. Therefore, a principal component analysis with varimax rotation was performed. The one-factorial solution explained 56% of the variance (*α* = 0.73). Participants rated the four items from 1 (not at all) to 5 (a lot). Sample items are “*I worry a lot*” (NEO-FFI) and “*I see myself as someone who is relaxed, handles stress well*” (reversed item, BFI-10).

FNE was assessed with the German Fear of Negative Evaluation Scale (SANB-5; Kemper et al., 2011). Participants rated five items from 1 (almost never applies) to 5 (almost always applies). A sample item is *“I fear doing or saying the wrong thing”*. Reliability was *α* = 0.88.

Social overload was measured with a six item-subscale (*α* = 0.74) of the Trier Inventory for the Assessment of Chronic Stress (TICS; Schulz et al., 2004). Items could be rated from 1 (never) to 5 (very often). A sample item is *“I constantly must care for others’ well-being anew.”*.

Occupational problems were assessed with a subscale of the Burnout Screening Scales assessing occupational problems (BOSS I; Hagemann & Geuenich, 2009). Ten items were rated from 1 (does not apply) to 6 (applies strongly). A sample item is *“I have lost the joy of my work”*. Reliability was *α* = 0.91 at t0, *α* = 0.83 at t1 and *α* = 0.83 at t2.

#### Behavioral observation

Four lessons of 45 min each were recorded with GoPro cameras and microphones in each class (after a three-day acclimatization period to reduce potential reactivity). The video material was coded and analyzed by four trained external observers in an event-sampling procedure using the BASYS observation system (Wettstein, 2008) with MAXQDA 2020 (VERBI Software, 2019). The BASYS observation system allows for coding disruptive behavior in school settings. Observed classroom disruptions included a working atmosphere marked by interruptions, lack of concentration, and restlessness. The observers and the teachers assessed the same class. Before coding, all four observers were trained to a criterion of 0.80 (Cohen’s kappa), and unclear episodes were discussed regularly. 11% of the coded video material was recoded, resulting in an inter-rater reliability of a Cohen’s kappa of 0.85. The level of observed classroom disruptions was determined continuously on a scale of one to four, with one indicating “hardly any disruptions” and four indicating “teaching hardly possible anymore”. Then, the relative frequency (in %) and the average of each of the four disruption levels were calculated for every teacher.

### Data analysis

For all scales, there were no missing values or imputations. The Shapiro–Wilk test was used for each variable to test normal distribution. Due to non-normal distribution, FNE, occupational problems at t0 and t1, and years of work experience were log-transformed. The observed classroom disruptions values were increased by 0.5 points to achieve semantic equivalence with the questionnaire for perceived classroom disruptions since the behavioral observation system and the teacher questionnaire were scaled semantically differently. All reported calculations used these transformed variables. Participants’ sex, years of work experience, and the number of lessons taught in the observed class were controlled for in the models when significantly related to the dependent or independent variable. The assumptions of multiple linear regression were tested and not violated, and multicollinearity diagnostics revealed correlations within an acceptable range.

All analyses were conducted using IBM SPSS Statistics (Version 28). The mediation was tested using an ordinary least square path analytic model with the PROCESS macro version 4.0 (Hayes, 2018). The significance of indirect effects was tested with a 95% confidence interval based on 5000 bias-corrected bootstrap samples. Descriptive statistics and bivariate correlations were computed for all variables to investigate hypotheses 1 and 2. Additionally, we computed differences between the perceived and observed classroom disruptions to descriptively explore teachers’ over- or underestimation. Teachers’ over- and underestimation is set in relation to observation and results from the discrepancy between teacher-perceived and observed classroom disruptions. Thus, overestimation refers to more teacher-perceived than observed classroom disruptions and underestimation to less teacher-perceived than observed classroom disruptions.

For hypothesis 3, we used a path-analytic mediation model with two parallel mediators to examine to which extent FNE and social overload mediated the effect of neuroticism on perceived classroom disruptions. To minimize the model’s complexity, control variables significantly correlated with perceived classroom disruptions were excluded as covariates.

To investigate predictors and consequences of teachers’ over- or underestimation of disruptions, we calculated polynomial regressions with response surface analysis (Shanock et al., 2010). This approach is useful regarding the teacher-perceived and observer rating discrepancy and its relation to an outcome. First, we examined if neuroticism, FNE, or social overload were related to an over- or underestimation (teacher-perceived and observer discrepancy) of classroom disruptions (hypothesis 4a). Second, we analyzed if an over- or underestimation of classroom disruptions was related to occupational problems at t0, t1, and t2 (hypothesis 4b).

Finally, we computed a multiple regression to examine how perceived classroom disruptions predict teachers’ occupational problems longitudinally (hypothesis 5).

## Results

### Descriptive statistics and bivariate correlations

Means, standard deviations, and bivariate Pearson correlations between observed and perceived classroom disruptions, neuroticism, FNE, social overload, occupational problems at t0, t1, and t2, and control variables are presented in Table 1.

**Table 1:** Descriptive statistics and intercorrelations

Variable	1	2	3	4	5	6	7	8	9	10
1. Observed classroom disruptions	–									
2. Perceived classroom disruptions	.27	–								
3. Neuroticism	.04	.39*	–							
4. FNE	.01	.47**	.45**	–						
5. Social overload	.11	.49**	.50**	.30	–					
6. Occupational problems t0	.28	.49**	.55**	.42**	.59**	–				
7. Occupational problems t1	.15	.58**	.62**	.47**	.33*	.59**	–			
8. Occupational problems t2	.19	.67**	.58**	.31	.37*	.46**	.76**	–		
9. Work experience	− .18	− .32*	− .01	− .21	− .09	− .02	− .05	− .10	–	
10. Lessons class	.14	.45**	.13	.16	.13	.12	.26	.24	− .28	–
11. Sex^a^	− .37*	− .15	− .10	− .01	− .06	− .06	− .04	− .29	.25	− .20
*M*	2.59	2.27	2.35	1.53	1.93	17.14	16.72	17.59	13.35	20.10
*SD*	2.89	0.61	0.74	0.53	0.63	7.63	4.84	5.79	11.07	5.11

t0: *N* = 42, t1 & t2: *N* = 39

^*^p < .05, **p < .01, two-tailed

^a^Sex: 0 = female; 1 = male

Observed and perceived classroom disruptions showed only a weak, non-significant association. Moreover, less experienced teachers reported more perceived classroom disruptions, and teachers with more lessons in the observed class experienced more perceived classroom disruptions. Classes of female teachers had more observed classroom disruptions.

Teachers’ neuroticism, FNE, social overload, and occupational problems were positively associated with perceived but not observed classroom disruptions.

### Over- and underestimation of classroom disruptions

First, teachers’ over- and underestimation of classroom disruptions defined as the discrepancy between observed (*Mdn* = 2.50) and teacher-perceived (*Mdn* = 2.25) ratings were analyzed. On average, teachers underestimated the classroom disruptions by 0.32 points (scale from 1 to 4). This difference was significant (Wilcoxon-test: *z* = −3.11, asymptotic *p* = 002, *n* = 42). The effect size is *r* = 0.47 and corresponds to a large effect (Cohen, 1992). While eleven teachers (26.19%) overestimated the disruptions (on average by 0.45 points), one teacher (2.38%) reported the same level of disruptions as observed, and 30 teachers (71.43%) underestimated the disruptions (on average by 0.60 points).

### Mediation analysis

**Fig. 1:**
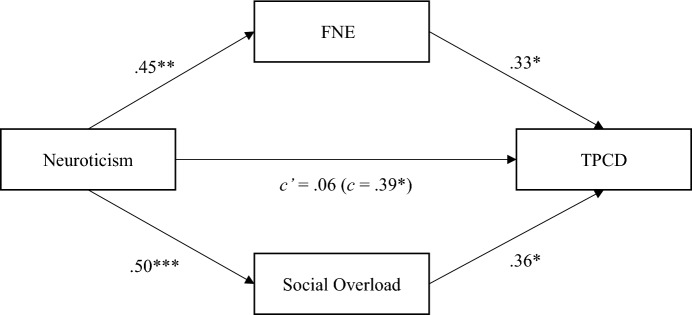
Mediation between neuroticism and teacher-perceived classroom disruptions with fear of negative evaluation and social overload as mediators. *Note N* = 42, FNE = fear of negative evaluation, TPCD = teacher-perceived classroom disruptions, *c’* = direct effect, *c* = total effect, coefficients are standardized. Indirect effect FNE: β = .15, 95% CI[0.02, 0.30], indirect effect social overload: β = .33, 95% CI[0.05, 0.36]. *p < .05, **p < .01, ***p < .001

A mediation model with two parallel mediators revealed that both FNE and social overload mediate the effect of neuroticism on perceived classroom disruptions (Fig. 1).

While the total effect of neuroticism on teacher-perceived classroom disruptions was significant (β = 0.39, *p* = 0.011), the direct effect was not (β = 0.06, *p* = 0.728). Overall, FNE and social overload mediated the relationship between neuroticism and teacher-perceived classroom disruptions, resulting in a total indirect effect of β = 0.33, 95% CI: 0.24 to 0.99. First, neuroticism had a significant effect on teacher-perceived classroom disruptions through FNE (β = 0.15, 95% CI: 0.02 to 0.30), indicating that teachers high in neuroticism more likely reported high FNE and, through high levels of FNE, more likely perceived more classroom disruptions. Second, there was a significant indirect effect through social overload (β = 0.33, 95% CI: 0.05 to 0.36), such that teachers high in neuroticism were more likely to experience social overload and, thus, more likely to report more perceived classroom disruptions.

### Polynomial regressions with response surface analysis

Polynomial regressions with response surface analysis were conducted to test hypothesis 4a, with perceived and observed classroom disruptions as independent, work experience, total lessons taught in class, and sex as control variables, and neuroticism, FNE, and social overload as dependent variables (Table 2). The regression results for neuroticism show that the coefficient of perceived classroom disruptions was significant (*p* = .010). Table 4 (see Appendix) shows that the estimated values for neuroticism are lowest on the congruence line (perceived = observed classroom disruptions). The more teachers over- or underestimate classroom disruptions, the higher their neuroticism values. Especially when the disruptions get overestimated, the estimated neuroticism values are the highest.

**Table 2:** Polynomial regression results for the effects of perceived and observed classroom disruptions on neuroticism, FNE and social overload

Variable	N	FNE	SO
Intercept	.842**	.546	2.156***
Control variables			
Work experience	.035	− .034	.041
Lessons class	− .003	− .003	− .009
Sex	− .067	.014	.010
Predictors			
PCD	.291*	.281**	.606**
OCD	− .187	− .286	− .293
PCD^2^	.105	.031	.016
PCD × OCD	− .415	− .253	− .900
OCD^2^	.190	.562	.446
*R*^*2*^	.22	.27	.30
CS	.104	− .005	.313
CC	− .120	.340	− .438
IS	.478	.567*	.899
IC	.710	.846	1.362

*N* = 42, all estimates are unstandardized, *N* = neuroticism, *FNE* = fear of negative evaluation, *SO* = social overload, *OCD* = observed classroom disruptions, *PCD = * perceived classroom disruptions, *R*^2^ = coefficient of determination, *CS* = the slope of the congruence line (OCD = PCD), *CC* = the curvature of the congruence line, *IS* = the slope of the incongruence line (OCD = − PCD), *IC* = the curvature of the incongruence line

^*^p < .05, **p < .01, ***p < .001

For FNE, the regression results show a significant coefficient (*p* = .009) for perceived classroom disruptions. Furthermore, the slope of the incongruence line becomes significant (*p* = .038), indicating that the relationship between disagreement and FNE is additive linear. Table 5 (see Appendix) shows that the estimated values for FNE are lowest when the level of perceived and observed classroom disruptions was medium. When teachers overestimated the disruptions, the estimated FNE values were particularly high.

The regression results for social overload show a significant coefficient (*p* = .004) for perceived classroom disruptions. Table 6 (see Appendix) shows that on the congruence line, the estimated values for social overload are lowest. The higher the social overload values, the more the teachers under- or overestimate the disruptions. Specifically, the estimated social overload values are the highest when the disruptions get overestimated.

Polynomial regressions with response surface analysis were conducted to test hypothesis 4b, with perceived and observed classroom disruptions as independent, work experience, total lessons taught in class, sex, and occupational problems at t0 (if necessary) as control variables, and occupational problems at t0, t1, and t2 as dependent variable (Table 3). For occupational problems at t0, the regression results show a significant coefficient for perceived classroom disruptions (*p* = .006) and the slope of the congruence line (*p* = .037). The significant slope of the congruence line indicates that if teachers’ perception is congruent with observation for low classroom disruptions, the occupational problems are lowest. If it is congruent for high classroom disruptions, occupational problems are highest (as visible in Table 7 in Appendix).

**Table 3:** Polynomial regression results for the effects of perceived and observed classroom disruptions on occupational problems at t0, t1 and t2

Variable	OCt0	OCt1	OCt2
Intercept	2.829***	1.873***	15.798*
Control variables			
Work experience	.055	.027	.873
Lessons class	− .007	.004	− .12
Sex	.025	.014	− 3.275
Occupational problems t0	–	.294*	1.862
Predictors			
PCD	.343**	.173	6.330***
OCD	.233	− .100	.167
PCD^2^	.041	− .028	.225
PCD × OCD	− .111	− .056	6.479
OCD^2^	− .052	.072	− 7.513
*R*^2^	.30	.46	.56
CS	.576*	.032	6.497
CC	− .122	− .012	− .809
IS	.110	.273	6.163
IC	.100	.100	− 13.767

*N* = 42, t1 & t2: *N* = 39, all estimates are unstandardized, *OC* = occupational problems, *OCD* = observed classroom disruptions, *PCD* = perceived classroom disruptions, *R*^2^ = coefficient of determination, *CS* = the slope of the congruence line (OCD = PCD), *CC* = the curvature of the congruence line, *IS* = the slope of the incongruence line (OCD = − PCD), *IC* = the curvature of the incongruence line

^*^p < .05, **p < .01, ***p < .001

Only the coefficient of occupational problems at t0 (for control) is significant (*p* = .029) for the regression results of occupational problems at t1. Table 8 (see Appendix) illustrates that both the congruence and incongruence lines show linearly increasing values for higher occupational problems with more and more perceived and observed classroom disruptions.

The regression results for occupational problems at t2 revealed a significant coefficient for perceived classroom disruptions (*p* < .001). Table 9 (see Appendix) shows that the congruence line tends to linearly increase in occupational problems values when perceived and observed classroom disruptions also increase. Furthermore, the estimated occupational problems values are higher when teachers overestimate classroom disruptions and lower when they underestimate them.

### Longitudinal associations of classroom disruptions with occupational problems

A multiple regression examined the association between perceived and observed classroom disruptions with occupational problems at t0 [*F*(5, 36) = 2.99, *p* = .003, *R*^2^_adjusted_ = 0.20]. While perceived disruptions significantly predicted occupational problems at t0 (β = .53, *p* = .003), observed disruptions did not (β = .18, *p* = .249), when controlling for work experience (β = .16, *p* = .316), lessons taught in observed class (β = −.09, *p* = .571), and sex (β = .03, *p* = .862).

The longitudinal analysis revealed that perceived disruptions were significantly associated with occupational problems at t2 (β = .66, *p* < .001) when controlling for occupational problems at t0 (β = .12, *p* = .450), work experience (β = .11, *p* = .425), and lessons taught in class [β = −.04, *p* = .781; *F*(4, 34) = 7.65, *p* < .001, *R*^2^_adjusted_ = 0.41].

## Discussion

This study aimed to compare classroom disruptions perceived by teachers and rated by external observers. Furthermore, risk factors such as neuroticism, FNE and social overload potentially influencing teachers’ perception of classroom disruptions were investigated. Moreover, consequences of teacher-perceived classroom disruptions on occupational problems were examined over two years.

Hypothesis 1, that teacher-perceived and observed classroom disruptions are only weakly to moderately correlated, was confirmed. Associations between teacher-perceived and observed classroom disruptions were weak and non-significant. This replicates findings from other studies (Scherzinger & Wettstein, 2019; Skiba, 1989) and suggests that teachers do not perceive classroom disruptions in an objective way. By further exploring the differences between teacher and observer perspective, descriptive analyses showed that most teachers are too optimistic: In relation to observed classroom disruptions, most teachers significantly underestimated the occurrence of classroom disruptions. Thus, most teachers seem to look at their classroom environment through rose-tinted glasses. However, a negativity bias was also frequent, whereas a realistic estimation of classroom disruptions was rare. This is consistent with Wubbels et al. (1992), who found that most teachers are overly optimistic and overestimate the quality of their interpersonal behavior in comparison to student ratings, while a smaller proportion of teachers underestimate it.

Hypothesis 2, that teacher characteristics are associated with teacher-perceived but not with observed classroom disruptions was supported. While teacher-perceived classroom disruptions significantly correlated with neuroticism, FNE, and social overload, observed classroom disruptions did not. This is consistent with studies that found an increased perception of student misbehavior for emotionally labile teachers (Kokkinos et al., 2005) and a biased perception and more psychological stress for individuals reporting high FNE and social overload (Maier et al., 2012; Zhang et al., 2016, 2022).

Explanations for teachers’ underestimation of classroom disruptions and for teacher characteristics influencing their perception could lie in how teachers attribute and elaborate their classroom environment. First, whether the construct has a positive or negative connotation; second, whether the construct refers to manifest behavior or not; third, whether the teachers’ attribution is internal or external; fourth, whether the teacher believes that the event is controllable or not; fifth, whether the attribution is neuronally fast or slow.

Classroom disruptions are negatively connotated, manifest, and observable events occurring in the classroom, and from the teacher’s point of view, they are attributed mostly to the students. Neurotic teachers may often attribute classroom disruptions externally as student disruptions (i.e., negative events) because neurotic individuals might more often externally attribute negative events and view them as less controllable (Caci et al., 2020; Darvill & Johnson, 1991). Teachers generally underestimate the occurrence of classroom disruptions in their classrooms. Such self-serving attributions are neuronally fastest, whereas internal attributions of negative events are slowest (Seidel et al., 2010). With high social density, simultaneity, and immediacy in the classroom, rapid, self-serving external attributions to classroom disruptions are probably most efficient, especially for socially overloaded teachers.

Hypothesis 3, that FNE and social overload mediate the effect of neuroticism on teacher-perceived classroom disruptions, was confirmed. The indirect effect of FNE and the indirect effect of social overload in the mediation model were significant. These results support the assumption that neuroticism increases teachers’ perception of classroom disruptions indirectly through FNE and social overload. Therefore, with FNE and social overload, our study sheds light on one so far unknown mechanism of the previously found association between neuroticism and student misbehavior (Kokkinos, 2007). Furthermore, this suggests targeting FNE and social overload by suitable interventions.

Hypothesis 4 assumed that an overestimation of classroom disruptions is positively associated with neuroticism, FNE, social overload, as well as occupational problems over the course of two years. Our results showed that neuroticism biased teachers’ perception of classroom disruptions in such a way that higher neuroticism values were positively associated with an over- as well as underestimation of classroom disruptions. The same pattern was found for FNE and social overload, indicating that both risk factors are more pronounced when teachers and observers do not agree. Moreover, these effects were stronger for overestimation, supporting hypothesis 4a. This showed that teachers high in neuroticism, FNE, and social overload are overestimating the disruptions occurring in their classroom.

Moreover, our results showed that an overestimation of classroom disruptions was associated with more occupational problems at t0 and t2, partially supporting hypothesis 4b.

Hypothesis 5, that perceived but not observed classroom disruptions are associated with occupational problems over the course of two years, was supported. Correlational data showed moderate to strong correlations between occupational problems at t0, t1, and t2 and teacher-perceived classroom disruptions, but not observed disruptions. Moreover, our longitudinal analysis suggests that the association between perceived classroom disruptions and occupational problems persists over the course of two years. The results even show that perceived classroom disruptions relate to an accumulation of occupational problems over two years. That suggests that occupational problems have a recursive effect on teachers’ perception. A vicious cycle might evolve in which the perception of classroom disruptions becomes more and more maladaptive, and occupational problems play an increasingly important role. This is in line with the transdisciplinary stress model of Epel et al. (2018), in which chronic stress can greatly impact the likelihood of developing maladaptive acute stress processes.

It can be assumed that risk factors such as neuroticism, FNE, and social overload have far-reaching consequences on teachers’ perception of classroom disruptions. These factors seem to promote an overestimation of disruptions, subsequently leading to more occupational problems. Thus, at-risk teachers might perceive their classroom environment as increasingly hostile. Their response to these perceived disruptions might become maladaptive, which in turn can worsen their relationship with students and their ability to manage their classroom effectively. This potentially creates a tiring cycle of biased perception and an increasingly disruptive environment to which teachers themselves contribute. This corresponds to Maslach and Jackson’s (1981) three burnout dimensions of depersonalization, reduced personal performance, and emotional exhaustion.

In line with this reasoning, a study by Kokkinos et al. (2005) showed that teachers’ level of emotional exhaustion affected their appraisal of the severity of students’ externalizing behavior. Chang and Davis (2009) showed that depending on their judgment about student behavior, teachers may use counterproductive approaches when faced with disruptive behavior, which leads to conflict escalation and emotional depletion. Consequently, it is less crucial how many disruptions actually occur in the classroom than how teachers perceive disruptive events in the classroom. The underlying processes in the relationship between personality and perception are mediated by FNE and social overload. Therefore, it is essential to sensibilize teachers to personal risk factors influencing their perception.

### Implications

Classroom disruptions are a primary source of teacher stress and endanger teachers’ health. Our study shows that teachers’ occupational problems are not associated with classroom disruptions per se but with how teachers perceive disruptions and that this perception also has a long-term impact. Furthermore, teachers’ perception is influenced by teacher characteristics such as neuroticism, FNE, and social overload. Therefore, teacher education must focus on teachers’ strategies for dealing with FNE, social overload, and attention steering when facing classroom disruptions. Regarding FNE, such strategies could facilitate distancing oneself from negative evaluations and facing them more serenely. Regarding social overload, such strategies could mean more recovery from work and a stronger segmentation of work and non-work boundaries (Allen et al., 2014). Teachers should be encouraged to view and evaluate classroom stressors as teaching challenges and to learn to manage stress actively (Chan, 1998). It is critical to sensitize teachers to potential strategies to prevent them from leaving the teaching profession early. In this way, burnout rates and costs can be kept lower, and the quality of the education system is not jeopardized.

### Limitations and strengths

The presented findings are limited to a small sample of apparently healthy and medication-free teachers. Thus, the results should not be generalized to the entire teacher population. Second, we examined the effects of social overload on classroom disruptions. However, classroom disruptions could also influence social overload, questioning the causal direction. Third, FNE was less prevalent among the teachers from our sample compared to a representative non-clinical German sample (Kemper et al., 2012), probably due to the social-interactional demands of the profession. Fourth, the sample consists of healthy teachers, and therefore, neuroticism is not strongly pronounced (Rammstedt, 2007; representative German sample).

A considerable strength of the present study is the combination of self-reports and behavioral observations in an ambulatory setting, improving our understanding of teachers’ perception of classroom disruptions compared to that of observers and potential reasons for their low agreement. Another strength of the study is its longitudinal design, which increases our comprehension of long-term consequences and suggests targets for interventions.

Future research should examine a broader range of factors that might influence teachers’ perception of the classroom environment and what implications this has for other health outcomes of teachers.

## Conclusion

This study shows that teacher-perceived and observed classroom disruptions are weakly and not significantly associated. Teacher characteristics such as neuroticism, FNE, and social overload influence teachers’ perception. Perceived classroom disruptions are associated with neuroticism, whereas this relationship is mediated by FNE and social overload. Therefore, combining self-reports with behavioral data has the potential to shed new light on the processes underlying teacher perception. Furthermore, perceived classroom disruptions are positively associated with more occupational problems two years later. Neuroticism, FNE, and social overload influence teachers’ perception in the form of over- and underestimation of classroom disruptions. Additionally, an over- and underestimation of classroom disruptions is positively associated with occupational problems at the first measurement point and two years later.

## Figures and Tables

**Table 4:**
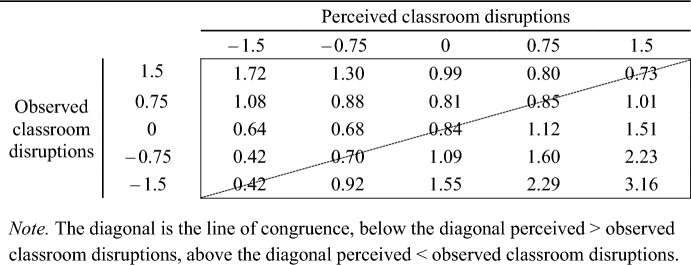
Predicted values of neuroticism for each combination of the two predictor variables

**Table 5:**
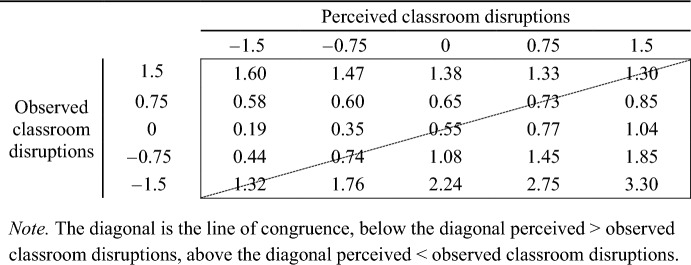
Predicted values of FNE for each combination of the two predictor variables

**Table 6:**
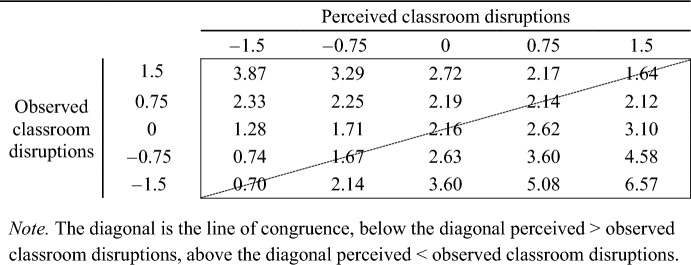
Predicted values of social overload for each combination of the two predictor variables

**Table 7:**
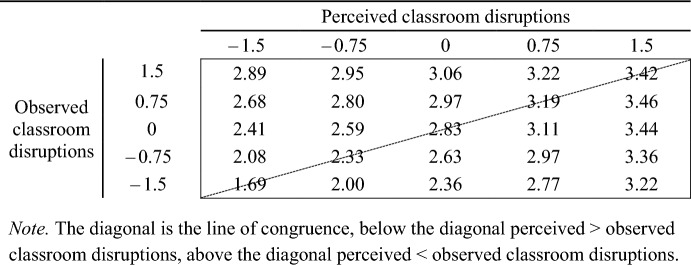
Predicted values of occupational problems t0 for each combination of the two predictor variables

**Table 8:**
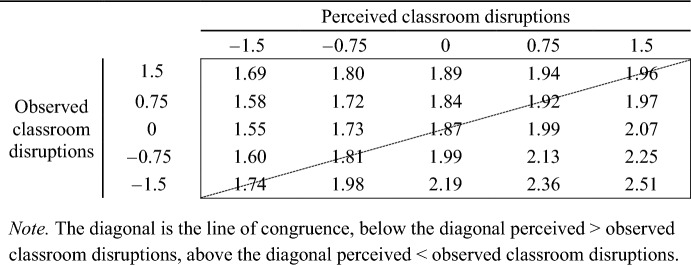
Predicted values of occupational problems t1 for each combination of the two predictor variables

**Table 9:**
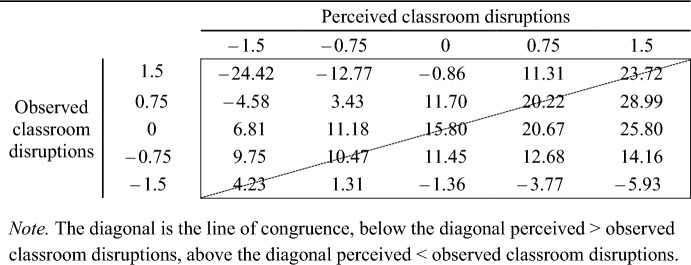
Predicted values of occupational problems t2 for each combination of the two predictor variables

## Data Availability

Data is available upon reasonable request from the corresponding author.

## Appendix

See Tables 4, 5, 6, 7, 8 and 9.
